# SARS-CoV-2 lateral flow assays for possible use in national covid-19 seroprevalence surveys (React 2): diagnostic accuracy study

**DOI:** 10.1136/bmj.n423

**Published:** 2021-03-02

**Authors:** Maya Moshe, Anna Daunt, Barnaby Flower, Bryony Simmons, Jonathan C Brown, Rebecca Frise, Rebecca Penn, Ruthiran Kugathasan, Claire Petersen, Helen Stockmann, Deborah Ashby, Steven Riley, Christina Atchison, Graham P Taylor, Sutha Satkunarajah, Lenny Naar, Robert Klaber, Anjna Badhan, Carolina Rosadas, Federica Marchesin, Natalia Fernandez, Macià Sureda-Vives, Hannah Cheeseman, Jessica O’Hara, Robin Shattock, Gianluca Fontana, Scott J C Pallett, Michael Rayment, Rachael Jones, Luke S P Moore, Hutan Ashrafian, Peter Cherapanov, Richard Tedder, Myra McClure, Helen Ward, Ara Darzi, Paul Elliott, Graham S Cooke, Wendy S Barclay

**Affiliations:** 1 Department of Infectious Disease, Imperial College London, School of Medicine, St Mary’s Hospital, Praed Street, London W2 1NY, UK; 2 Imperial College Healthcare NHS Trust, St Mary’s Hospital, London, UK; 3 School of Public Health, Imperial College London, St Mary’s Hospital, London, UK; 4 Institute for Global Health Innovation, Imperial College London, London, UK; 5 Synthetic Biology Group, London Institute of Medical Sciences, Imperial College London, London, UK; 6 Chelsea and Westminster NHS Foundation Trust, London, UK; 7 Centre of Defence Pathology, Royal Centre for Defence Medicine, Queen Elizabeth Hospital, Birmingham, UK; 8 Department of Surgery and Cancer, Imperial College London, London, UK; 9 Chromatin Structure and Mobile DNA Laboratory, Francis Crick Institute, London, UK; 10 National Institute for Health Research (NIHR) Health Protection Research Unit (HPRU) in Chemical and Radiation Threats and Hazards, Imperial College London, London, UK

## Abstract

**Objective:**

To evaluate the performance of new lateral flow immunoassays (LFIAs) suitable for use in a national coronavirus disease 2019 (covid-19) seroprevalence programme (real time assessment of community transmission 2—React 2).

**Design:**

Diagnostic accuracy study.

**Setting:**

Laboratory analyses were performed in the United Kingdom at Imperial College, London and university facilities in London. Research clinics for finger prick sampling were run in two affiliated NHS trusts.

**Participants:**

Sensitivity analyses were performed on sera stored from 320 previous participants in the React 2 programme with confirmed previous severe acute respiratory syndrome coronavirus 2 (SARS-CoV-2) infection. Specificity analyses were performed on 1000 prepandemic serum samples. 100 new participants with confirmed previous SARS-CoV-2 infection attended study clinics for finger prick testing.

**Interventions:**

Laboratory sensitivity and specificity analyses were performed for seven LFIAs on a minimum of 200 serum samples from participants with confirmed SARS-CoV-2 infection and 500 prepandemic serum samples, respectively. Three LFIAs were found to have a laboratory sensitivity superior to the finger prick sensitivity of the LFIA currently used in React 2 seroprevalence studies (84%). These LFIAs were then further evaluated through finger prick testing on participants with confirmed previous SARS-CoV-2 infection: two LFIAs (Surescreen, Panbio) were evaluated in clinics in June-July 2020 and the third LFIA (AbC-19) in September 2020. A spike protein enzyme linked immunoassay and hybrid double antigen binding assay were used as laboratory reference standards.

**Main outcome measures:**

The accuracy of LFIAs in detecting immunoglobulin G (IgG) antibodies to SARS-CoV-2 compared with two reference standards.

**Results:**

The sensitivity and specificity of seven new LFIAs that were analysed using sera varied from 69% to 100%, and from 98.6% to 100%, respectively (compared with the two reference standards). Sensitivity on finger prick testing was 77% (95% confidence interval 61.4% to 88.2%) for Panbio, 86% (72.7% to 94.8%) for Surescreen, and 69% (53.8% to 81.3%) for AbC-19 compared with the reference standards. Sensitivity for sera from matched clinical samples performed on AbC-19 was significantly higher with serum than finger prick at 92% (80.0% to 97.7%, P=0.01). Antibody titres varied considerably among cohorts. The numbers of positive samples identified by finger prick in the lowest antibody titre quarter varied among LFIAs.

**Conclusions:**

One new LFIA was identified with clinical performance suitable for potential inclusion in seroprevalence studies. However, none of the LFIAs tested had clearly superior performance to the LFIA currently used in React 2 seroprevalence surveys, and none showed sufficient sensitivity and specificity to be considered for routine clinical use.

## Introduction

A detailed understanding of severe acute respiratory syndrome coronavirus 2 (SARS-CoV-2) seroprevalence is key to public health policy and in anticipating the epidemiology of the coronavirus disease 2019 (covid-19) pandemic. In contrast to routine serology assays, the use of lateral flow immunoassays (LFIAs) does not require the support of central laboratories and offers a rapid, scalable, and affordable method of testing. This approach has been used in Spain^1^ and in our own React 2 (real time assessment of community transmission 2) study in England^2^ to conduct national seroprevalence studies^2^ and to monitor the persistence of SARS-CoV-2 antibodies.^3^ The React 2 programme consists of participants self-administering LFIAs at home. Participants complete an online questionnaire and read the results by using uploaded test images.^2^ The first round of the programme consisted of more than 100 000 participants and was completed on 13 July 2020. The results showed a SARS-CoV-2 antibody prevalence of approximately 6% nationally, with ethnic minority groups, healthcare workers, and care home workers disproportionately affected.^2^ Two subsequent rounds of surveillance have now been completed, all using the same assay (Fortress Diagnostics, Northern Ireland), which was selected after rigorous clinical and laboratory evaluation^4^ and engagement with the public and participants.^5^

Diagnostics development continues at pace internationally, with over 200 LFIAs commercialised.^6^ Despite this effort, no LFIA evaluated to date meets the Medicines and Healthcare products Regulatory Agency criteria for approval for individual testing in the United Kingdom,^7^ which requires the sensitivity (proportion of people with SARS-CoV-2 infection with a positive test result) and specificity (proportion of people without SARS-CoV-2 infection with a negative test result) to exceed 98%. When used in population studies, analyses can adjust for the performance characteristics of tests that do not meet such stringent criteria. However, to ensure these adjustments are accurate, evaluation in the intended setting of use is required. At present, the evaluation of LFIAs has focused on performance in the laboratory.^8^ ^9^ Rigorous clinical evaluation of SARS-CoV-2 LFIAs of different population subgroups (such as people admitted to hospital compared with those not admitted to hospital, or people with severe symptoms compared with those without symptoms) is urgently needed to enhance generalisability and reduce variability in sensitivity and specificity estimates.^10^

In this study, we continue our programme of evaluating LFIAs that have been prioritised from published evaluations and that have the potential for large scale application. The primary objective was to establish the sensitivity and specificity of new LFIAs, and to identify the most suitable candidate for deployment in future rounds of the React 2 study and potentially for individual use. A new LFIA would be considered to replace the Fortress LFIA in future rounds of React 2 seroprevalence surveys if it showed significantly superior sensitivity on finger prick testing and the capacity to be procured rapidly and at scale.

## Methods

The methods reported here describe round 2 of our LFIA evaluation (React 2, study 1) and are based on the same principles as the round 1 methods.^4^ However, in contrast to round 1, LFIAs were only evaluated on capillary blood in the clinic if they showed equivalent or superior sensitivity and specificity on testing of sera in the laboratory compared with the Fortress LFIA currently being used for the React 2 seroprevalence studies. A comprehensive React protocol has been published and describes the study design, sampling size and strategy, and data collection and analysis of the various ongoing React 1 and React 2 studies.^11^

### Laboratory assessment of sensitivity and specificity using sera

Initial assessment of LFIA sensitivity used sera stored from 320 participants from round 1 with a previous positive test for SARS-CoV-2 by reverse transcription polymerase chain reaction (RT-PCR) on nasopharyngeal swab. Supplementary figure i presents a detailed summary of the flow of participants during sensitivity and specificity analysis in the laboratory. Sera were stored at −80ºC, subjected to a maximum of two freeze-thaw cycles, and brought to room temperature before testing. Research technicians blinded to the reference standard assay results performed LFIA testing in the laboratory. The two reference standards used in this study—the in-house SARS-CoV-2 spike protein enzyme linked immunoassay (S-ELISA) and a hybrid double antigen binding assay (hybrid DABA)—have been shown to have high sensitivity and specificity; these reference standards have been described previously.^4^ The viral antigen used in the S-ELISA is the SARS-CoV-2 spike protein whereas the hybrid DABA uses the SARS-CoV-2 spike protein and receptor binding domain. This variation results in inherent differences in the detection of antibody classes between the two in-house ELISAs; therefore, a composite outcome of a positive result on S-ELISA or hybrid DABA was used as the benchmark for sensitivity analysis throughout this study. Research technicians performing LFIA testing in the laboratory were aware of whether they were testing LFIAs for sensitivity or specificity, but were blinded to clinical information about participants and the reference standard assay. Assessors of the reference standard were blinded to clinical information and LFIA test results, and repeated any borderline positive or negative samples to give a determinate result.

Sensitivity in the laboratory for detection of SARS-CoV-2 antibodies was estimated for each LFIA using a minimum of 200 sera and compared with positive results on the S-ELISA or hybrid DABA. When testing the AbC-19 LFIA, a scoring card supplied by the manufacturer was used to grade the intensity of immunoglobulin G (IgG) bands on a scale of 1-10 (supplementary table i). All other LFIA results were interpreted as either IgG positive or negative. Specificity analysis was performed on prepandemic sera collected as part of the Airwave Health Monitoring Study before August 2019.^12^ Round 2 used a different cohort of 500 prepandemic sera (Airwave2) from that used previously (Airwave1).^4^

### Test selection for clinic

**Fig 1:**
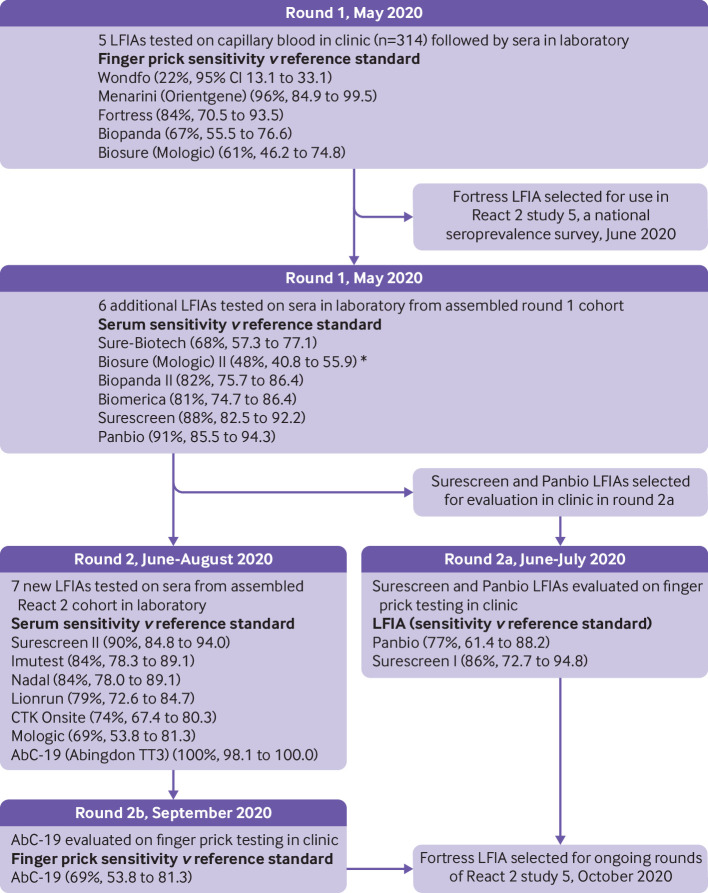
Timeline and selection process for lateral flow immunoassay (LFIA) evaluation. Further information about flow of participants is included in supplementary figures i and ii. *Biosure (Mologic) II was tested with 5 µL serum (according to instructions provided at time). Manufacturer advises LFIA should be performed with 10 µL serum. React 2=real-time assessment of community transmission 2

In round 1 of the React 2 programme,^4^ five LFIAs were initially evaluated using finger prick testing in the clinic. These LFIAs also underwent sensitivity and specificity analyses on sera from the assembled cohort and 500 prepandemic sera, respectively. A further six LFIAs underwent sensitivity analysis and four of these achieved sufficient sensitivity to proceed to specificity testing on 500 prepandemic sera. In round 1, specificity was performed on LFIAs that showed a sensitivity of more than 80%. The two best performing LFIAs, which also showed high specificity scores of 99.8% (Surescreen, Panbio), were identified for potential clinic evaluation in round 2 (fig 1).

In this study, we report the results of round 2 of the study in which seven further LFIAs were initially selected for evaluation based on the manufacturer’s performance and published data, if available (supplementary table ii), before undergoing sensitivity analysis. LFIAs with a laboratory sensitivity greater than the sensitivity of the Fortress LFIA (>84%) proceeded to specificity testing on 500 prepandemic sera. The AbC-19 LFIA was selected for clinic evaluation because it showed the best performance in the laboratory and it has the potential to be procured at scale. Altogether, three LFIAs were selected for testing in the clinic in round 2: Panbio (Abbott^13^), AbC-19 (TT3, Abingdon rapid test consortium^14^), and Surescreen.^15^

### Participant recruitment in clinic

Clinical recruitment of participants took place over two periods. Round 2a ran from 17 June to 2 July 2020 and tested the Panbio and Surescreen tests in parallel in the same participants. Round 2b ran from 4 to 21 September 2020 and tested the AbC-19 LFIA. People who worked in one of five NHS hospitals in two NHS trusts were invited to participate. Additionally, participants who had been involved in previous rounds were invited to reattend to test new LFIAs. No participants tested the same LFIA more than once. Supplementary figure ii presents a detailed summary of the flow of participants during sensitivity evaluation in the clinic.

Eligibility criteria were broadened in round 2 (supplementary fig iii). People who were known to be or had been seropositive based on formal laboratory antibody testing performed before attending the study clinic, or people with PCR confirmed SARS-CoV-2 infection (or both) were included. Additionally, family members of staff could participate provided they had also received a positive PCR result or were previously confirmed to be seropositive on formal laboratory antibody testing. Finally, participants who were admitted to hospital with covid-19 and were previously excluded from the study could also take part. Participants were enrolled after a minimum of 21 days had passed from symptom onset or positive PCR result (whichever occurred earlier). People considered seropositive for SARS-CoV-2 antibodies on previous finger prick LFIA only were excluded.

### Clinic procedure

Study clinics were run at two sites in round 2a and at a single site in round 2b. Participants were required to provide evidence of the result and date of a previous positive SARS-CoV-2 PCR or laboratory based antibody test, and to complete a questionnaire. The questionnaire included demographic information, medical history, and information detailing the timing, duration, and severity of illness caused by SARS-CoV-2 infection.

Each participant performed one or two LFIAs using capillary blood through finger prick, under the supervision of a research nurse or practitioner, before analysis against reference standards. Participants followed the protocol provided by the manufacturer and verbal instructions from trained research staff in the clinic to ensure that the test was performed correctly. Interpretation of the LFIA result by the participant and trained observer was recorded independently and photographs of the completed tests were obtained. At each attendance, a venous blood sample was taken for laboratory testing. Invalid or failed tests, where the control line was absent, were excluded from the analysis and participants repeated the test.

To enable direct comparison of performance with capillary blood in clinic and sera in the laboratory, one LFIA (AbC-19) was retested by a research technician using matched sera from clinic participants, according to the manufacturer’s protocol (supplementary table i).

### Sample size

Sample size was calculated assuming 90% power, SARS-CoV-2 infection prevalence of 100%, and expected test sensitivity of 85%. To evaluate sensitivity with a two sided delta of 10%, a target sample size of 153 participants was calculated. For specificity, a sample size of 361 was calculated based on an expected specificity of 98% with a lower limit of 95%.

### Performance analysis

Statistical analysis was performed as previously described.^4^ The primary outcome of the study was the sensitivity and specificity of each LFIA in detecting SARS-CoV-2 IgG antibodies. Sensitivity analysis included performance on finger prick self-testing (participant interpretation), finger prick self-testing (observer interpretation), and serum in the laboratory. Two comparisons were made: against confirmed previous SARS-CoV-2 infection (by PCR swab or previous laboratory antibody test) and against confirmed positive results by S-ELISA or hybrid DABA from venous samples taken at the study clinic appointment. As previously described, specificity was calculated as the proportion of known negative samples that were negative on the LFIAs. Data are presented using a binomial confidence interval of 95% and significance was denoted by a P value less than 0.05.

For comparison of clinic and laboratory performance of individual LFIAs, agreement was assessed using the κ statistic with the following interpretation: less than 0, poor agreement; 0.00-0.20, slight agreement; 0.21-0.40, fair agreement; 0.41-0.6, moderate agreement; 0.61-0.8, good agreement; and higher than 0.8, almost perfect agreement^16^ Analysis of antibody concentrations is presented using quantitative S-ELISA data for round 2a and 2b. S-ELISA titres showed a skewed distribution and were log_10_ transformed. Continuous variables are presented as medians and interquartile ranges; the Kruskal-Wallis test was used to compare continuous variables across rounds. We used Dunn’s test to evaluate pairwise comparisons when the Kruskal-Wallis test was rejected. The McNemar test was used to test differences for dependent groups. We used Fisher’s exact test to test differences between independent groups. To further explore test sensitivity, participants were divided into quarters according to S-ELISA antibody reactivity, and test sensitivity was assessed for each category. Statistical analyses were performed on Stata (version 14.2, StataCorp, TX).

### Patient and public involvement

Public involvement and participant feedback have been central to the design of the React 2 programme. There has been extensive involvement from patient panels and rigorous evaluation of the usability of LFIAs included in the React 2 studies has been undertaken.^5^ User expressed feedback during clinics, and formal evaluation of instruction materials provided to manufacturers through patient panels have been incorporated in reports to companies. 

## Results

Table 1 and table 2 show full results for sensitivity and specificity testing of nine LFIAs tested in round 2 using sera in the laboratory. The Fortress LFIA, previously studied in round 1, was retested against the Airwave2 prepandemic sera and attained a specificity of 99.0% (95% confidence interval 97.7% to 99.7%). The overall specificity of Fortress LFIA was 98.8% (97.9% to 99.4%) against 1000 prepandemic serum samples. Table 1, table 2, and supplementary table iii present the sensitivity of the composite reference standards S-ELISA and hybrid DABA when tested on participants from round 1, 2a, and 2b.

**Table 1:** Sensitivity results for LFIAs tested on round 1, 2a, and 2b positive sera and specificity results for LFIAs tested on prepandemic negative sera. Data are percentages (95% confidence intervals)

LFIA	Cohort	Sensitivity with sera *v* reference standard*	n/N	Cohort	Sensitivity with finger prick test *v* reference standard*	n/N	Sensitivity with matched sera *v* reference standard*	n/N	Cohort	Specificity (95% CI)	n/N	Invalid (n)
**Assessed with serum and blood**												
AbC-19 (IgG only)	1, 2a, 2b	100 (98.1 to 100)	192/192	2b	69 (53.8 to 81.3)	33/48	92 (80.0 to 97.7)	44/48	2	99.8 (98.9 to 100)	499/500	4
Panbio (separate IgG and IgM)	1	91 (85.5 to 94.3)	173/191	2a	77 (61.4 to 88.2)	33/43	—	—	1	99.8 (98.9 to 100)	499/500	0
Surescreen (separate IgG and IgM)	1	88 (82.5 to 92.2)	168/191	2a	86 (72.7 to 94.8)	38/44	—	—	1	99.8 (98.9 to 100)	499/500	0
Fortress (separate IgG and IgM)	1	89 (84.3 to 92.0)	262/296	1	84 (70.5 to 93.5)	38/45	93 (81.7 to 98.6)	42/45	1, 2	98.8 (97.9 to 99.4)	988/1000	3
	—	—	—	2b	—	—	83† (69.8 to 92.5)	40/48	1	98.6 (97.1 to 99.4)	493/500	—
	—	—	—	—	—	—	—	—	2	99.0 (97.7 to 99.7)	495/500	—
**Assessed with serum only**												
Surescreen II (IgG alone)	1, 2a	90 (84.8 to 94.0)	164/182	—	—	—	—	—	2	100 (99.3 to 100)	500/500	0
Imutest (separate IgG and IgM)	1, 2a	84 (78.3 to 89.1)	161/191	—	—	—	—	—	2	100 (98.5 to 100)	250/250	0
Nadal (separate IgG and IgM)	1, 2a	84 (78.0 to 89.1)	154/183	—	—	—	—	—	—	—	—	0
Lionrun (separate IgG and IgM)	1, 2a	79 (72.6 to 84.7)	148/187	—	—	—	—	—	—	—	—	5
CTK onsite (separate IgG and IgM)	1, 2a	74 (67.4 to 80.3)	141/190	—	—	—	—	—	—	—	—	0
Mologic (separate IgG, IgM, and IgA)	1	69 (53.8 to 81.3)	33/48	—	—	—	—	—	—	—	—	0

DABA=double antigen binding assay; IgA=immunoglobulin A; IgG=immunoglobulin G; IgM=immunoglobulin M; LFIA=lateral flow immunoassay; S-ELISA=spike protein ELISA.

* Spike protein enzyme linked immunoassay or hybrid double antigen binding assay.

† Sensitivity of the Fortress lateral flow immunoassay with round 2b sera.

**Table 2:** Validation of positive and negative sera on reference standards. Data are percentages (95% confidence intervals)

Sera and cohort	Sensitivity or specificity *v* reference standard*	n/N
**Sensitivity analysis**		
Positive sera		
Cohort 1	96 (93.2 to 97.8)	307/320
Cohort 2a	95.8 (85.8 to 99.6)	46/48
Cohort 2b	96 (86.3 to 99.5)	46/48
**Specificity analysis**		
Airwave prepandemic sera		
Cohort 1	100 (99.3 to 100.0)	498/498

Validation of the Airwave prepandemic sera cohort 2 was performed on the Fortress lateral flow immunoassay and is included in table 1.

* Spike protein enzyme linked immunoassay or hybrid double antigen binding assay.

Forty six of 48 participants who attended clinic in round 2a, and 51 of 52 who attended clinic in round 2b underwent finger prick testing and were included in the analysis (supplementary fig ii). Table 3 and supplementary table iv present their baseline characteristics. The sensitivity of the Surescreen and Panbio LFIAs, prioritised from round 1, were 88% (82.5% to 92.2%) and 91% (85.5% to 94.3%), respectively on testing of sera, and both had 99.8% specificity (98.9% to 100%).

**Table 3:** Baseline characteristics of participants in round 1, 2a, and 2b (assessed on Fortress, Surescreen and Panbio, and AbC-19 lateral flow immunoassays, respectively). Data are medians (interquartile ranges) unless stated otherwise

Baseline characteristics	Round 1 (n=48)	Round 2a (n=46)	Round 2b (n=51)
**Participant characteristics**			
Age (years)	34 (28-47)	38 (30-47)	42 (33-52)
Women, n (%)	32 (67)	30 (65)	34 (67)
Role, n (%)			
Doctor	16 (33)	15 (33)	5 (10)
Nurse or midwife	20 (42)	9 (20)	19 (37)
Other clinical	4 (8)	6 (13)	9 (18)
Non-clinical	8 (17)	16 (35)	18 (35)
Covid-19 characteristics,* n (%)			
Asymptomatic	3 (6)	4 (9)	6 (12)
Mild	5 (10)	10 (22)	8 (16)
Moderate	27 (56)	16 (35)	18 (35)
Severe, not admitted to hospital	13 (27)	13 (28)	15 (29)
Severe, admitted to hospital	NA	3 (7)	4 (8)
**Method of covid-19 diagnosis, n (%)**			
PCR test and antibody positive	NA	1 (2)	3 (6)
PCR test	48 (100)	28 (61)	20 (39)
Laboratory based antibody test	NA	17 (37)	28 (55)
**Symptoms (days)**			
Duration of symptoms	14 (10-17)	11 (6-27)	13 (8-33)
Time since symptom onset	59 (49-69)	90 (80-101)	173 (165-181)
Time since positive test	47 (36-56)	74 (65-87)	108 (92-155)
**Reference standard results**			
Confirmed previous infection, n/N (%)	48/48 (100)	46/46 (100)	51/51 (100)
Positive S-ELISA, n/N (%)	45/48 (94)	46/46 (100)	48/50 (96)
Positive hybrid DABA, n/N (%)	43/48 (90)	33/37 (89)	41/50 (82)
S-ELISA† (log_10_ ng/mL)	3.1 (2.3-3.4)	3.3 (3.0-3.6)	2.7 (2.3-3.0)

covid-19=coronavirus disease 2019; DABA=double antigen binding assay; IQR=interquartile range; NA=not applicable; PCR=polymerase chain reaction; S-ELISA=spike protein enzyme linked immunoassay.

Percentages are calculated from non-missing values. For participants with ongoing symptoms, symptom duration was assessed as date of start of symptoms to date of visit. Time since symptom onset assessed for participants with symptoms only.

* Self-assessed disease severity.

† Includes participants positive on S-ELISA; 14 did not have a quantitative S-ELISA result available.

**Fig 2:**
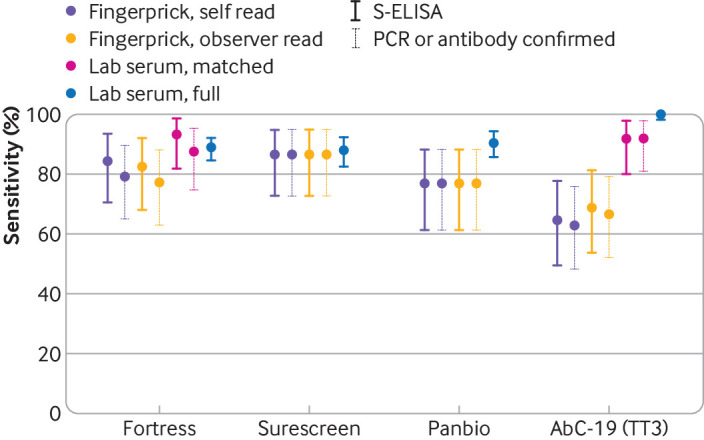
Sensitivity of lateral flow immunoassays for finger prick (self-read), finger prick (observer read), matched serum, and initial analysis with serum. S-ELISA data include sensitivity results versus the in-house SARS-CoV-2 S-ELISA; PCR or antibody confirmed data include sensitivity results versus previous confirmed SARS-CoV-2 infection by PCR or formal laboratory antibody test result. PCR=polymerase chain reaction; SARS-CoV-2=severe acute respiratory syndrome coronavirus 2; S-ELISA=spike protein enzyme linked immunoassay

The Surescreen LFIA tested in the clinic attained a sensitivity of 86% (72.7% to 94.8%) compared with the benchmark ELISAs, which was not significantly different (P=0.8) than it had shown against round 1 sera in the laboratory or significantly higher (P=0.772) than the Fortress LFIA. By contrast, AbC-19 and Panbio tests showed a lower sensitivity on finger prick capillary blood in the clinic compared with sera in the laboratory (table 1, table 2, and fig 2). The sensitivity of Panbio dropped significantly (P=0.018) from 91% (85.5% to 94.3%) in the laboratory with round 1 sera to 77% (61.4% to 88.2%) on finger prick blood in clinic versus S-ELISA or hybrid DABA.

Despite attaining the highest sensitivity (100%, 98.1% to 100%) during laboratory testing with stored sera in the laboratory, the sensitivity of AbC-19 LFIA with capillary blood through finger prick also reduced significantly (P<0.001) to 69% (53.8% to 81.3%) versus S-ELISA or hybrid DABA. Supplementary table v includes additional sensitivity estimates for each LFIA by method of SARS-CoV-2 diagnosis (PCR *v* previous formal laboratory antibody testing) and by symptom severity.

Given the possibility that the cohort tested on AbC-19 had low antibody titres, matched serum samples from the participants evaluated in the clinic were tested in the laboratory on the AbC-19 LFIA and a sensitivity of 92% (80.0% to 97.7%) was found. This result was significantly higher than the sensitivity on finger prick testing in the clinic performed on the same participants (P=0.007). Concordance between the sensitivity for finger prick and for sera from these matched samples, determined by κ score, was slight (0.07, 95% confidence interval −0.14 to 0.28) in contrast to moderate (0.56, 0.25 to 0.86) for matched samples previously tested on the Fortress LFIA.

Because of low rates of new SARS-CoV-2 infection locally at the time of round 2 clinic testing, we anticipated that a proportion of participants might have been infected at an early stage of the pandemic. The round 2b cohort had a significantly longer period since symptom onset of 173 days compared with 90 days for round 2a and 59 days for round 1 (table 3 and supplementary fig iii; P<0.001). To account for the possibility that waning of antibody titres had led to an underestimation of the AbC-19 LFIA sensitivity performance in clinic, the distribution of serum antibody concentrations was analysed for round 1, 2a, and 2b on S-ELISA (supplementary fig iii). The median antibody concentration on S-ELISA was significantly different for round 1, 2a, and 2b (P<0.001). Of samples with antibody titres in the lowest quarter (≤2.51 log_10_ S-ELISA or 325 ng/mL), the Fortress LFIA detected 8/12 positive samples on finger prick testing compared with 8/21 on AbC-19 (supplementary figs iv and v). Additionally, when tested using sera matched to participants in clinic, the AbC-19 LFIA detected 20/21 samples as opposed to 8/21 from the same participants tested by finger prick in clinic (supplementary table vi). Supplementary table vi presents sensitivity results for all antibody concentration quarters for the LFIAs tested on finger prick in clinic and matched sera (if applicable).

## Discussion

This study shows that LFIA sensitivity is variable on serum and finger prick testing, and often differs from that stated by the manufacturer. Specificity of all LFIAs that underwent this analysis was high. One further LFIA (Surescreen) is identified as suitable for use in seroprevalence studies because it showed comparable performance to the LFIA currently used in the React 2 seroprevalence studies (Fortress). However, the performance of Surescreen was not significantly better than Fortress; as a result, Fortress is still considered the most suitable candidate for ongoing rounds of the React 2 programme.

LFIAs remain an important tool in the assessment of population seroprevalence of SARS-CoV-2 infection. Evaluation of new LFIAs often relies on performance using sera in the laboratory with only a minority of studies evaluating whole blood or capillary finger prick testing, which ultimately is the intended use.^17^ An accurate assessment of the performance of LFIAs with capillary blood is key to interpreting large scale seroprevalence studies, and before clinical implementation, given the reduced sensitivity compared with laboratory analysis for some tests.^4^ ^18^

Laboratory testing is an essential component of LFIA evaluation.^15^ ^18^ In this study, as in previous work, we have shown that most LFIAs evaluated had lower sensitivity or specificity than reported in preliminary results by the manufacturers. Only three LFIAs in round 2, and eight of 18 LFIAs evaluated in the React 2 programme to date showed sufficient sensitivity and specificity during analysis on sera to justify progression to clinic testing.

Of the three LFIAs tested in the clinic in this study, two showed a significant difference in sensitivity between serum and finger prick testing in two tests. AbC-19, the best performing test in the laboratory (100% sensitivity, 95% confidence interval 98.1% to 100.0%; and 99.8% specificity, 98.9% to 100.0%), showed the lowest sensitivity (69%, 53.8% to 81.3%) compared with S-ELISA or hybrid DABA upon finger prick testing in the clinic. Of the remaining two tests, only the Surescreen LFIA showed a marginally higher sensitivity than the Fortress LFIA, which has been used in previous rounds of the React national seroprevalence study.

Several potential reasons could explain differences in test performance between the laboratory and clinic. A possibility is that with sequential testing, participants recruited later after acute infection are likely to have lower antibody titres. Time elapsed post symptom onset was considerably different between all three rounds. Additionally, by broadening the inclusion criteria to include those with positive serology only (as opposed to PCR positivity), later cohorts might represent milder disease or be more likely to contain participants with false positive results. However, these more varied presentations of previous SARS-CoV-2 infection are a more accurate reflection of the population for which the LFIAs are intended to be used than healthcare employees testing positive on previous PCR alone.

Distribution of antibody titres was substantially different across all three periods of testing. A rapid fall in new SARS-CoV-2 infections locally after the first wave of the pandemic, and the timing at which new LFIAs became available for evaluation meant that there was a considerable difference in time since symptom onset in rounds 1, 2a, and 2b. Therefore, we considered whether a possible threshold effect relating to antibody concentration could account for a drop off in sensitivity observed between laboratory and clinic testing. However, on evaluation of the lowest quarter of serum antibody concentration from samples across all three rounds, the Fortress LFIA detected a numerically higher proportion of samples with a low antibody titre on finger prick testing than AbC-19. Additionally, the median antibody titre in round 2a was higher than that of round 1; despite this, the Panbio LFIA also showed a (non-significant) difference in sensitivity between serum and finger prick testing. Finally, the considerable difference in sensitivity on the AbC-19 LFIA between finger prick and sera testing on matched samples from round 2b suggests other factors in test design and use could be more important.

### Study limitations

Several limitations are acknowledged in this study. Important differences were found between cohorts and corresponding antibody titres. Additionally, while laboratory staff were blinded to the results of LFIA interpretation on finger prick testing, participants and research staff in the clinic were aware that all participants had evidence of previous SARS-CoV-2 infection. Therefore, LFIA interpretation in the clinic was not blinded, which could have led to an overestimation of LFIA sensitivity on self-testing. In round 2a, participants were evaluated on two LFIAs in the same appointment and it is possible that the result of the test evaluated first could have influenced the interpretation of the second test.

### Conclusions

This study confirms the importance of assessing LFIAs in the intended population because laboratory results might not accurately predict performance in the clinic. We have shown that analyses should account for changes in antibody levels over time and the comparison of tests on a consistent cohort of laboratory sera remains an important part of the evaluation. Through a robust approach to LFIA evaluation, we characterised the performance of nine LFIAs, and identified one new LFIA with performance comparable to the Fortress LFIA, which has been used successfully in large seroprevalence studies. For our React 2 programme, a new LFIA would have to perform considerably better than the Fortress LFIA to outweigh the scientific value in repeating seroprevalence surveys using the same assay. At this time, no LFIA offers enough improvement in performance to merit inclusion in subsequent React 2 seroprevalence studies. Additionally, no LFIA has reached the standard set by the Medicines and Healthcare products Regulatory Agency for individual use.

**What is already known on this topic?**

Since the start of the coronavirus disease 2019 (covid-19) pandemic, over 200 lateral flow immunoassays (LFIAs) have been developed to detect severe acute respiratory syndrome coronavirus 2 (SARS-CoV-2) antibodies
Performance of these LFIAs is variable
Previous studies have focused on laboratory performance, not on capillary blood testing, which is the intended use

**What this study adds**

The LFIAs analysed showed high specificity, but variable sensitivity on sera and finger prick testing
One new LFIA was found to be suitable for use in seroprevalence studies, but none of the LFIAs tested meet the criteria for individual use

## Data Availability

Anonymised data are available on reasonable request and can be provided by the corresponding author.

## Web extra

Extra material supplied by authors

Web appendix: supplementary files

## Notes

Contributors: All listed authors made substantial contributions to the conception or design of the work; or the acquisition, analysis, or interpretation of data for the work; and drafting the work or revising it critically for important intellectual content; final approval of the version to be published; and agreement to be accountable for all aspects of the work in ensuring that questions related to the accuracy or integrity of any part of the work are appropriately investigated and resolved. MM and AD contributed equally to this work; GSC and WSB contributed equally to this work. The corresponding author attests that all listed authors meet authorship criteria and that no others meeting the criteria have been omitted. GSC is the guarantor.

The lead author (the manuscript’s guarantor) affirms that the manuscript is an honest, accurate, and transparent account of the study being reported; that no important aspects of the study have been omitted; and that any discrepancies from the study as planned (and, if relevant, registered) have been explained.

Dissemination to participants and related patient and public communities: Once published, the results of this study will be circulated to Imperial College Healthcare and Chelsea and Westminster NHS staff who participated in the study. The results of the study will be provided to all participants who request to see them.

Provenance and peer review: Not commissioned; externally peer reviewed.

## References

[1] Pollán M Pérez-Gómez B Pastor-Barriuso R ENE-COVID Study Group . Prevalence of SARS-CoV-2 in Spain (ENE-COVID): a nationwide, population-based seroepidemiological study. Lancet 2020;396:535-44. https://linkinghub.elsevier.com/retrieve/pii/S0140673620314835. 10.1016/S0140-6736(20)31483-5 32645347 PMC7336131

[2] Ward H Atchinson JC Whitaker M . Antibody prevalence for SARS-CoV-2 in England following first peak of the pandemic: REACT2 study in 100 000 adults. MedRxiv. 2020. https://www.medrxiv.org/content/10.1101/2020.08.12.20173690v2 10.1101/2020.08.12.20173690

[3] Ward H Cooke G Atchinson C . Declining prevalence of antibody positivity to SARS-CoV-2: a community study of 365 000 adults. medRxiv. 2020

[4] Flower B, Brown JC, Simmons B, et al. Clinical and laboratory evaluation of SARS-CoV-2 lateral flow assays for use in a national COVID-19 seroprevalence survey. 2020. https://thorax.bmj.com/lookup/doi/10.1136/thoraxjnl-2020-215732 10.1136/thoraxjnl-2020-215732 PMC7430184 32796119

[5] Atchinson C, Pristerà P, Cooper E, et al. Usability and acceptability of home-based self-testing for SARS-CoV-2 antibodies for population surveillance. 2020. http://fdslive.oup.com/www.oup.com/pdf/production_in_progress.pdf

[6] FIND. SARS-CoV-2 diagnostic pipeline. 2020. https://www.finddx.org/covid-19/pipeline/

[7] MHRA. TARGET PRODUCT PROFILE Antibody tests to help determine if people have immunity to SARS-CoV-2. 2020. https://assets.publishing.service.gov.uk/government/uploads/system/uploads/attachment_data/file/883897/Target_Product_Profile_antibody_tests_to_help_determine_if_people_have_immunity_to_SARS-CoV-2_Version_2.pdf

[8] Silveira MF Barros AJD Horta BL . Population-based surveys of antibodies against SARS-CoV-2 in Southern Brazil. Nat Med 2020;26:1196-9. https://www.nature.com/articles/s41591-020-0992-3. 10.1038/s41591-020-0992-3 32641783

[9] Sood N Simon P Ebner P . Seroprevalence of SARS-CoV-2-specific antibodies among adults in Los Angeles County, California, on April 10-11, 2020. JAMA 2020;323:2425-7. https://jamanetwork.com/journals/jama/fullarticle/2766367. 10.1001/jama.2020.8279 32421144 PMC7235907

[10] Lisboa Bastos M Tavaziva G Abidi SK . Diagnostic accuracy of serological tests for covid-19: systematic review and meta-analysis. BMJ 2020;370:m2516. 10.1136/bmj.m2516 32611558 PMC7327913

[11] Riley S Atchison C Ashby D . REal-time Assessment of Community Transmission (REACT) of SARS-CoV-2 virus: study protocol. Wellcome Open Research. 2020. 10.12688/wellcomeopenres.16228.1. PMC8095190 33997297

[12] Elliott P Vergnaud AC Singh D Neasham D Spear J Heard A . The Airwave Health Monitoring Study of police officers and staff in Great Britain: rationale, design and methods. Environ Res 2014;134:280-5. 10.1016/j.envres.2014.07.025. 25194498

[13] Batra R Olivieri LG Rubin D . A comparative evaluation between the Abbott Panbio™ COVID-19 IgG/IgM rapid test device and Abbott Architect™ SARS CoV-2 IgG assay. J Clin Virol 2020;132:104645. https://linkinghub.elsevier.com/retrieve/pii/S1386653220303875. 10.1016/j.jcv.2020.104645 32961429 PMC7493757

[14] Robertson JL, Moore SJ, Blighe K, Ng YK, Quinn N, Jennings F, et al. SARS-CoV-2 antibody testing in a UK population: detectable IgG for up to 20 weeks post infection. medRxiv. 2020.

[15] Pickering S Betancor G Galão RP . Comparative assessment of multiple COVID-19 serological technologies supports continued evaluation of point-of-care lateral flow assays in hospital and community healthcare settings. PLoS Pathog 2020;16:e1008817. https://dx.plos.org/10.1371/journal.ppat.1008817. 10.1371/journal.ppat.1008817 32970782 PMC7514033

[16] Landis JR Koch GG . The measurement of observer agreement for categorical data. Biometrics 1977;33:159-74. https://www.jstor.org/stable/2529310?origin=crossref. 10.2307/2529310 843571

[17] Deeks JJ Dinnes J Takwoingi Y Cochrane COVID-19 Diagnostic Test Accuracy Group . Antibody tests for identification of current and past infection with SARS-CoV-2. Cochrane Database Syst Rev 2020;6:CD013652. 32584464 10.1002/14651858.CD013652 PMC7387103

[18] Conklin ES Martin K Manabe CY . Evaluation of serological SARS-CoV-2 lateral flow assays for rapid point of care test. medRxiv. 2020. 10.1128/JCM.02020-20 PMC8111122 33208477

