# Supplementary material for: SARS-CoV-2 lateral flow assays for possible use in national covid-19 seroprevalence surveys (React 2): diagnostic accuracy study
Source: BMJ. 2021 Mar 2;372:n423. doi: 10.1136/bmj.n423 (PMC7921617; doi:10.1136/bmj.n423)
Supplement: Supplementary file 1 — Web appendix: supplementary files [file mosm062782.ww.docx]

**SUPPLEMENTARY SECTION**

***Supplementary figure i:*** *Flow of participants in Sensitivity (left) and Specificity (right) evaluation with sera in the laboratory.*

**Specificity analysis on sera**

LFIAs with highest sensitivity on serum proceeded to specificity analysis on pre-pandemic sera (Airwaves Health Monitoring Study)

**Sensitivity analysis on sera**

Serum from participants from Round 1 cohort (n= 314) used to evaluate:

- Fortress (n=300)

- Panbio (n= 200)

- Surescreen (n=200)

- Mologic (n=50)

Sera from previous REACT-2 participants

500 pre-pandemic sera (Airwaves cohort 1) used to evaluate:

- Fortress (n=500)

- Panbio (n=500)

- Surescreen (n=500)

Further serum samples added to assembled cohort after completion of Round 2a clinic testing (n=48)

Sera from previous REACT-2 participants

Serum from participants from Round 1 and 2a (n= 362) used to evaluate:

- Surescreen II (n=200)

- Imutest (n=200)

- Nadal (n= 200)

- Lionrun (n=200)

- CTK onsite (n=200)

- AbC-19 (n=200)

Sera from previous REACT-2 participants

500 pre-pandemic sera (Airwaves cohort 2) used to evaluate:

- AbC-19 (n=500)

- Fortress additional specificity analysis (n=500)

- Surescreen II (n=500)

- Imutest (n=250)

***Supplementary figure ii:*** *Flow of participants in eligibility criteria and sensitivity analysis for finger-prick testing in clinic on participants in Rounds 2a (left) and 2b (right).*

**Finger-prick sensitivity analysis**

NHS staff members or family members invited to register interest via:

1. Email sent to all staff
2. Previous participants contacted directly by study team

**Eligible participants invited to research clinic**

**Inclusion Criteria**

- ≥ 18 years old
- Previous SARS-CoV-2 infection confirmed by PCR or laboratory antibody test
- ≥ 21 days since symptom onset

**Round 2a**

48 participants attended clinic

- 29 attending for 1st time

- 10 attending for 2nd time

- 9 attending for 3rd time

1 participant unable to be bled for venous sample

1 participant ineligible

50 participants provided venous blood for laboratory testing

48 participants provided venous blood for laboratory testing

51 participants underwent finger-prick testing

**Round 2b**

52 participants attended clinic

- 40 attending for 1st time

- 4 attending for 2nd time

- 5 attending for 3rd time

- 3 attending for 4^th^ time

46 participants underwent finger-prick testing

- 44 tested on Surescreen

- 43 tested on Panbio

2 participants excluded from fingerpick analysis due to missing data

***Supplementary figure iii:*** *A comparison of SARS-CoV-2 positive sera collected in three Rounds 1, 2a and 2b used in sensitivity analysis of lateral flow immunoassays in this study. The distribution of antibody titres for the three cohorts used in LFIA sensitivity analyses as measured on an in-house SARS-CoV-2 spike protein ELISA (S-ELISA) (left). A comparison of the elapsed time post symptom onset for Round 1, 2a and 2b significance for pairwise comparison between rounds (right). *p<0.05, **p<0.01, ***p<0.001.*

**
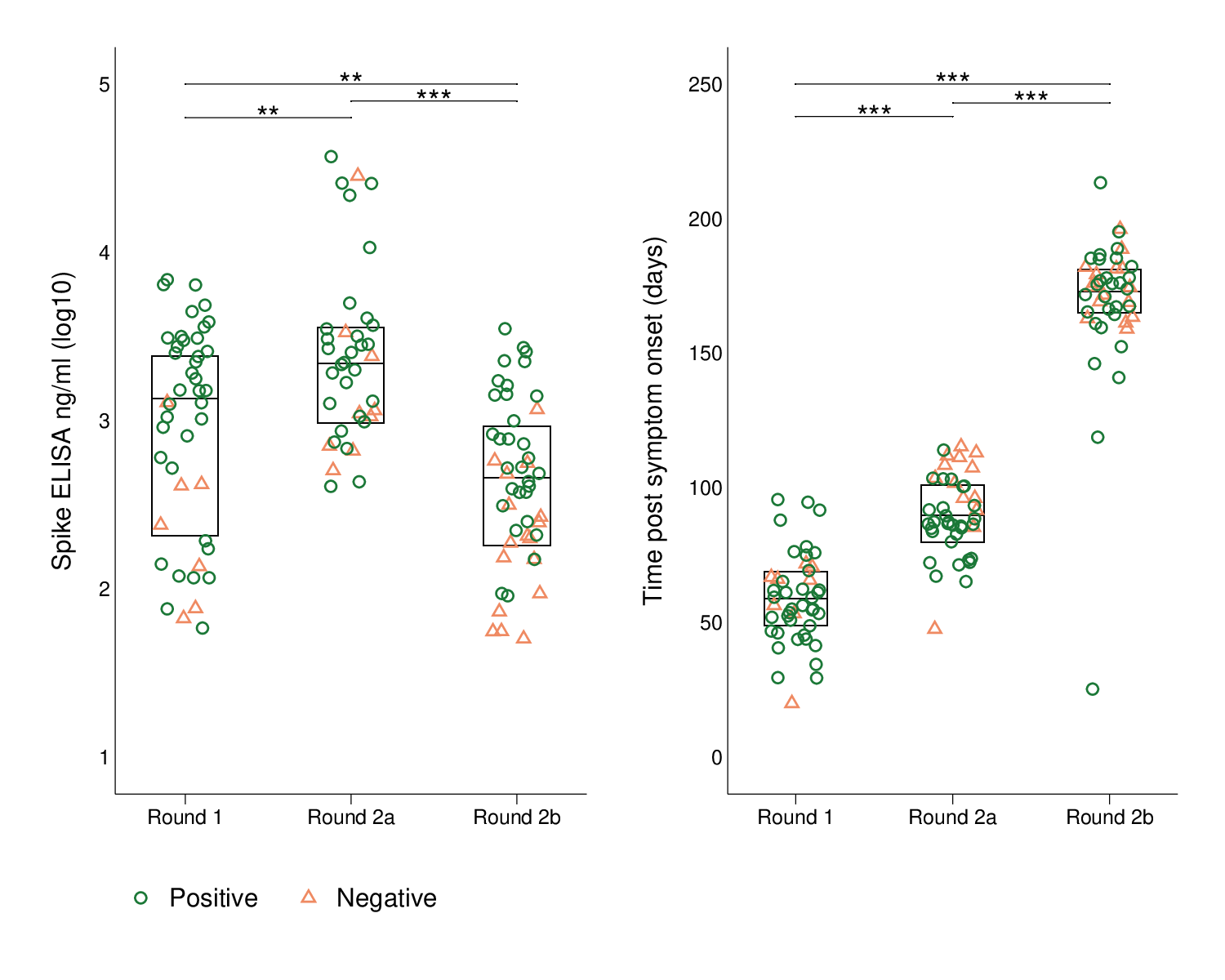
**

***Supplementary figure iv.*** *Sensitivity of two LFIAs on samples with antibody concentrations within the lowest quartile of quantitative S-ELISA results. This is compared between (top) Finger-prick (self-read), (middle) Finger-prick (observer-read) and (bottom) Matched serum. Sensitivity results are shown against S-ELISA (reference standard) and confirmed previous infection of SARS-CoV-2 by PCR and/or formal laboratory antibody test.*

**
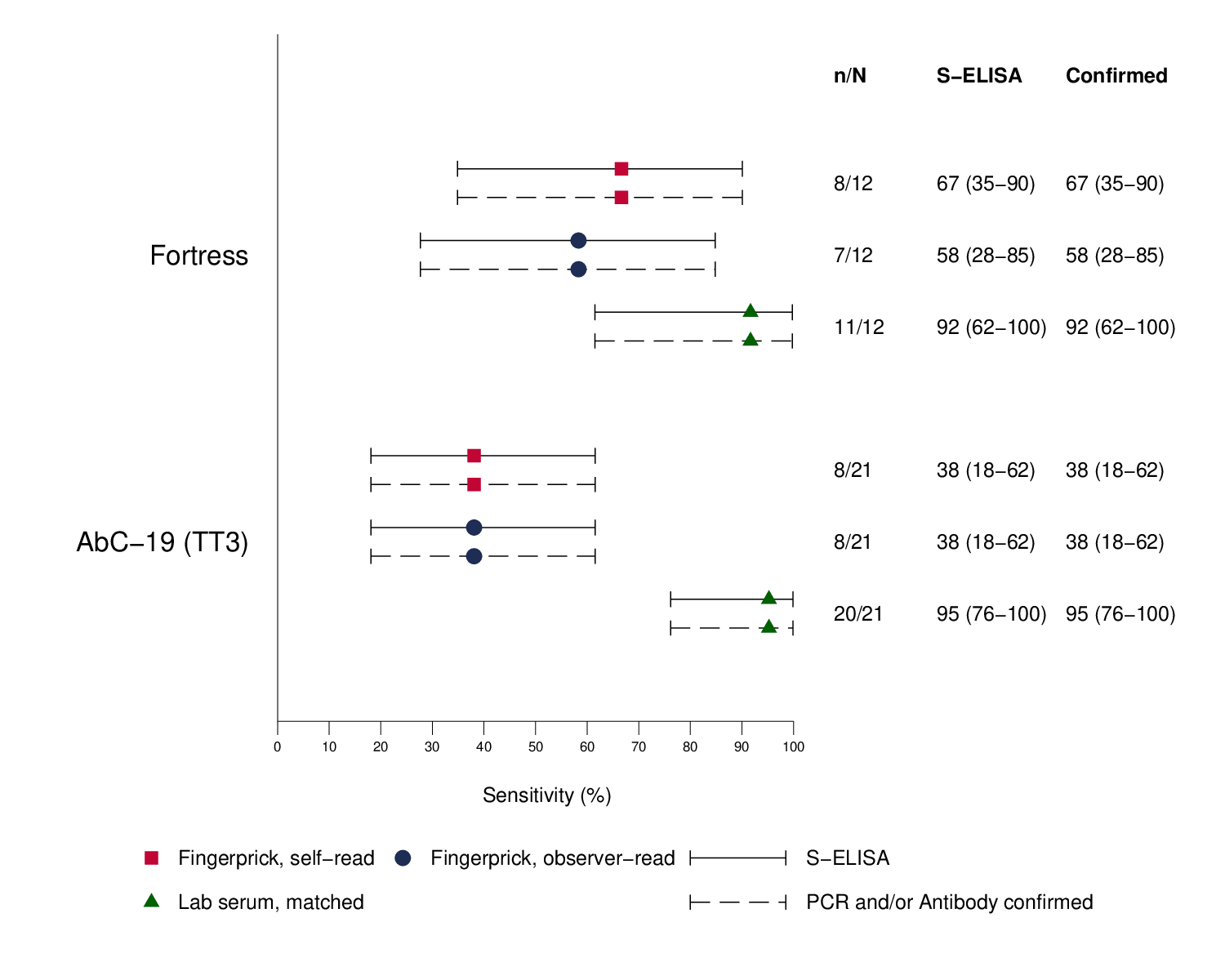
**

***Supplementary figure v:*** *Sensitivity on finger-prick testing of four LFIAs vs S-ELISA across different antibody titres by quartile. Sensitivities shown if calculation includes 5 or more results (n>5).*

**
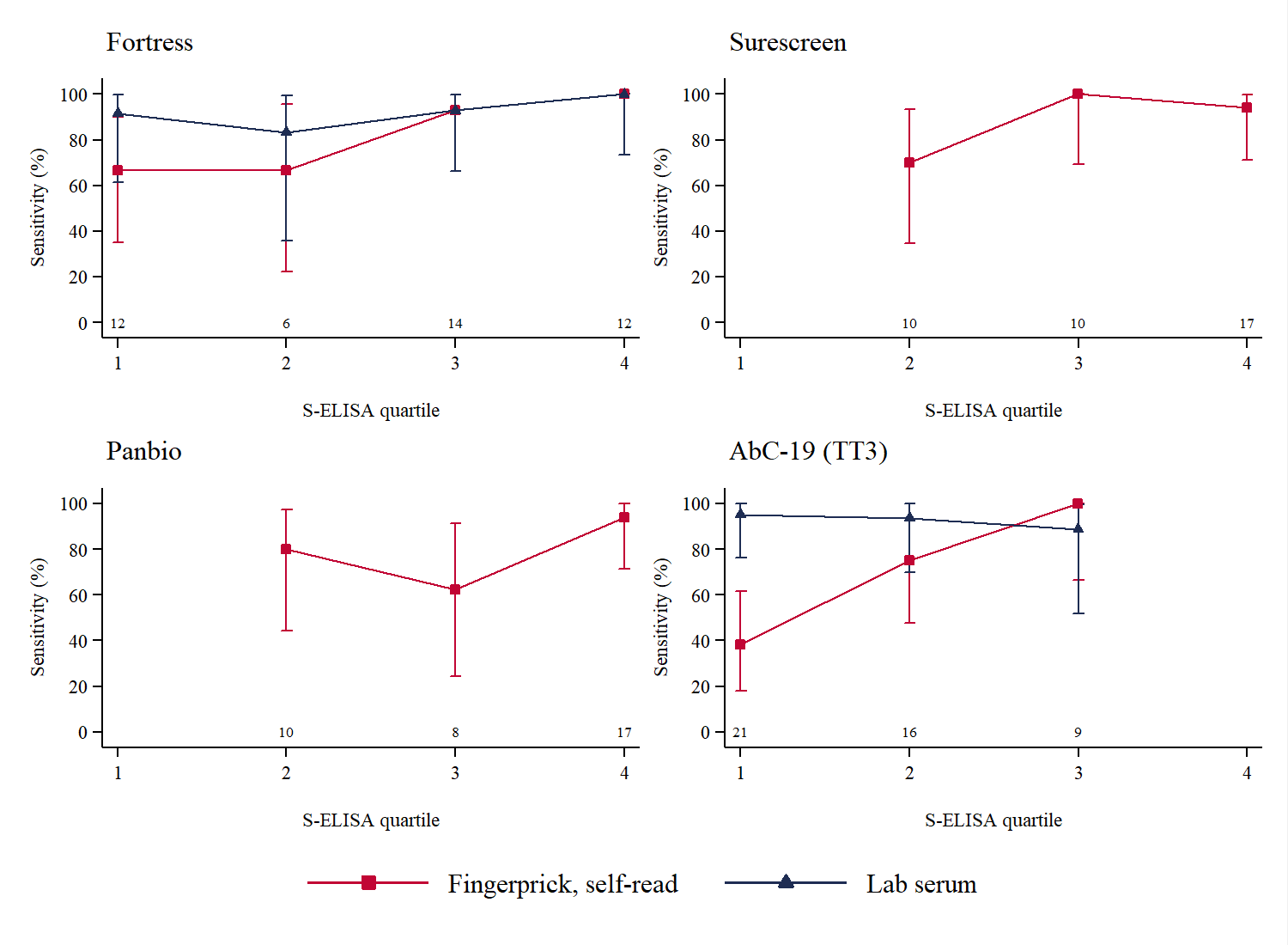
**

***Supplementary table i.*** *Comparison of band Intensity score for the AbC-19 LFIA when tested with capillary blood in the clinic versus with matched serum in the laboratory. A score of 0 indicates a negative result whereas a score of ≥1 indicates a positive result.*

| CAPILLARY BLOOD IN CLINIC | SCORE | SERUM IN LAB | SCORE |
| --- | --- | --- | --- |
| NEGATIVE | 0 | POSITIVE | 2 |
| NEGATIVE | 0 | POSITIVE | 2 |
| NEGATIVE | 0 | POSITIVE | 1 |
| NEGATIVE | 0 | POSITIVE | 1 |
| NEGATIVE | 0 | POSITIVE | 1 |
| NEGATIVE | 0 | POSITIVE | 1 |
| NEGATIVE | 0 | POSITIVE | 1 |
| NEGATIVE | 0 | POSITIVE | 1 |
| NEGATIVE | 0 | POSITIVE | 1 |
| NEGATIVE | 0 | NEGATIVE | 0 |
| NEGATIVE | 0 | POSITIVE | 1 |
| NEGATIVE | 0 | POSITIVE | 1 |
| NEGATIVE | 0 | POSITIVE | 1 |
| NEGATIVE | 0 | POSITIVE | 3 |
| NEGATIVE | 0 | POSITIVE | 2 |
| NEGATIVE | 0 | POSITIVE | 1 |
| NEGATIVE | 0 | NEGATIVE | 0 |
| POSITIVE | 2 | POSITIVE | 3 |
| POSITIVE | 1 | POSITIVE | 3 |
| POSITIVE | 2 | POSITIVE | 2 |
| POSITIVE | 2 | POSITIVE | 3 |
| POSITIVE | 1 | POSITIVE | 2 |
| POSITIVE | 2 | POSITIVE | 2 |
| POSITIVE | 2 | POSITIVE | 3 |
| POSITIVE | 6 | POSITIVE | 5 |
| POSITIVE | 2 | POSITIVE | 1 |
| POSITIVE | 4 | POSITIVE | 2 |
| POSITIVE | 1 | POSITIVE | 1 |
| POSITIVE | 1 | POSITIVE | 1 |
| POSITIVE | 1 | POSITIVE | 2 |
| POSITIVE | 2 | POSITIVE | 2 |
| POSITIVE | 2 | POSITIVE | 1 |
| POSITIVE | 2 | NEGATIVE | 0 |
| POSITIVE | 2 | POSITIVE | 2 |
| POSITIVE | 2 | POSITIVE | 1 |
| POSITIVE | 6 | POSITIVE | 1 |
| POSITIVE | 2 | POSITIVE | 1 |
| POSITIVE | 1 | NEGATIVE | 0 |
| POSITIVE | 2 | POSITIVE | 1 |
| POSITIVE | 5 | POSITIVE | 1 |
| POSITIVE | 1 | POSITIVE | 1 |
| POSITIVE | 2 | POSITIVE | 1 |
| POSITIVE | 4 | POSITIVE | 1 |
| POSITIVE | 2 | POSITIVE | 2 |
| POSITIVE | 4 | POSITIVE | 2 |
| POSITIVE | 1 | POSITIVE | 2 |
| POSITIVE | 6 | POSITIVE | 3 |
| POSITIVE | 4 | POSITIVE | 3 |
| POSITIVE | 2 | POSITIVE | 2 |
| POSITIVE | 2 | POSITIVE | 2 |

***Supplementary table ii:*** *Lateral flow immunoassay product specifications and manufacturer-reported performance.*

| **LFIA** | **PRODUCT SPECIFICATIONS** | **MANUFACTURER REPORTED SENSITIVITY (vs PCR-confirmed) – (IgG)** | **MANUFACTURER REPORTED SPECIFICITY (IgG)** |
| --- | --- | --- | --- |
| **FORTRESS**  Fortress Diagnostics COVID-19 TOTAL Ab Device, COVID010/COVID020. Separate IgG and IgM | Add 5-10μL of whole blood, serum or plasma into the specimen window. Immediately add two drops of diluent buffer into the buffer window. Read result 15m after specimen and buffer loading. | 95.6% (90.7 to 98.4) (n=137) | 95.2% (91.4 to 97.7) (n=209) |
| **SURESCREEN**  SureScreen Diagnostics LTD, COVID199GG  Separate IgG and IgM | Add approximately 10μL of whole blood, serum or plasma to the specimen well. Add 2 drops of buffer (approximately 80μl) to the buffer well. Read result after 10 minutes | 97.4% (86.2 to 99.9)  (n=38) | 98.9% (96.1 to 99.9)  (n=143) |
| **SURESCREEN II**  Surescreen Diagnostics LTD  COVID199GG IgG Only | Add approximately 10μL of whole blood, serum or plasma to the specimen well. Add 2 drops of buffer (approximately 80μl) to the buffer well. Read result after 10 minutes. | 96.50% (92.3 to 98.6)  (n=200) | >99% (verbally confirmed, awaiting final data as per instruction manual) |
| **PANBIO (ABBOTT)**  Panbio COVID-19 IgG/IgM Rapid Test ICO-T40203  Separate IgG and IgM | Add 20μL of whole blood or 10μl of serum or plasma to specimen well. Add 2 drops of buffer. Read result after 10 minutes | Finger-prick 96.2%  Venous whole blood: 96.0%  Plasma 97.7%  (n not specified) | Finger-prick: 100.0  Venous whole blood: 95.8%  Plasma 92.8%  (n not specified) |
| **AbC-19 (TT3, ABINGDON UK RAPID CONSORTIUM),** FG-FD51919  IgG only | Add 2.5μL of whole blood or serum to the sample well and immediately add the buffer provided or 100uL buffer when using serum. Read result in 20 minutes. | 98.03% (95.03 to 99.46%) | 99.56% (98.40 to 99.95%) |
| **NADAL**  NAL VON MINDEN, Moers, Germany  Separate IgG and IgM | Add 10μl of whole blood, serum or plasma into to the first sample well. Add 2 drops of buffer (approximately 80μL of buffer) into the buffer | 94.1% (n and 95% CI not specified) | 99.2% |
| **LIONRUN**  Shanghai Liangrun Biomedicine Technology Co. LTD, DMID/DE/0000047791  Separate IgG and IgM | Add 20μl of whole blood or 10μl of serum or plasma to the sample well of the test cassette. Add 2-3 drops of buffer into the buffer well. Read test after 10 minutes | 90.4% (85.3 to 94.2)  (n=188) | 100% (98.0 to 100.0)  (n=182) |
| **CTK ONSITE**  CTK Biotech, California, USA, R0180C  Separate IgG and IgM | Add 20-30μl of whole blood, or 10-15μl of serum or plasma to the specimen well. Add 3 drops of buffer (approximately 100μl) to the buffer well. Read result at 10-20minutes. | 97.1% (n not specified) | 97.8% (n not specified) |
| **MOLOGIC**  Mologic COVID-19 blood IgA/IgG/IgM rapid diagnostic test (RDT)  AUG 2020 (VERSION 4; REF 11911125). | Apply 5uL of blood/serum/plasma to round sample well and ensure the droplet is absorbed. Apply 2 drops of chase buffer to the round sample well. Read result after 10 minutes. | 94.02% (91.0 to 96.3) (n=351) | 99.2% (CI 97.96 to 99.69) (n=603) |

***Supplementary table iii:*** *LFIA sensitivity compared to PCR and/or antibody confirmed results, S-ELISA and hybrid DABA.*

| **Lateral flow assay** | **Test method** | | | **Positive, n/N(%)** | | **Positive vs S-ELISA, n/N(%)** | **Positive vs DABA, n/N(%)** | | **Sensitivity against references standards, % (95% CI)** | | | |
| --- | --- | --- | --- | --- | --- | --- | --- | --- | --- | --- | --- | --- |
|  |  |  |  |  |  |  |  |  | PCR/Antibody confirmed | | S-ELISA | Hybrid DABA |
|  |  | | |  | |  |  | |  | |  |  |
| Fortress | Finger-prick, self-read | | | 38/48 (79) | | 38/45 (84) | 38/43 (88) | | 79 (65.0 to 89.5) | | 84 (70.5 to 93.5) | 88 (74.9 to 96.1) |
|  | Finger-prick, observer-read | | | 37/48 (77) | | 37/45 (82) | 37/43 (86) | | 77 (62.7 to 88.0) | | 82 (67.9 to 92.0) | 86 (72.1 to 94.7) |
|  | Lab serum | | | 42/48 (88) | | 42/45 (93) | 41/43 (95) | | 88 (74.8 to 95.3) | | 93 (81.7 to 98.6) | 95 (84.2 to 99.4) |
|  |  | | |  | |  |  | |  | |  |  |
| Surescreen | Finger-prick, self-read | | | 38/44 (86) | | 38/44 (86) | 29/32 (91) | | 86 (72.6 to 94.8) | | 86 (72.6 to 94.8) | 91 (75.0 to 98.0) |
|  | Finger-prick, observer-read | | | 38/44 (86) | | 38/44 (86) | 29/32 (91) | | 86 (72.6 to 94.8) | | 86 (72.6 to 94.8) | 91 (75.0 to 98.0) |
|  | Lab serum | | |  | |  |  | |  | |  |  |
|  |  | | |  | |  |  | |  | |  |  |
| Panbio | Finger-prick, self-read | | | 33/43 (77) | | 33/43 (77) | 25/30 (83) | | 77 (61.4 to 88.2) | | 77 (61.4 to 88.2) | 83 (65.3 to 94.4) |
|  | Finger-prick, observer-read | | | 33/43 (77) | | 33/43 (77) | 26/30 (87) | | 77 (61.4 to 88.2) | | 77 (61.4 to 88.2) | 87 (69.3 to 96.2) |
|  | Lab serum | | |  | |  |  | |  | |  |  |
|  |  | | |  | |  |  | |  | |  |  |
| AbC-19 | Finger-prick, self-read | | | 32/51 (63) | | 31/48 (65) | 28/41 (68) | | 63 (48.1 to 75.9) | | 65 (49.5 to 77.8) | 68 (51.9 to 81.9) |
|  | Finger-prick, observer-read | | | 34/51 (67) | | 33/48 (69) | 30/41 (73) | | 67 (52.1 to 79.2) | | 69 (53.7 to 81.3) | 73 (57.1 to 85.8) |
|  | Lab serum | | | 46/50 (92) | | 44/48 (92) | 37/41 (90) | | 92 (80.8 to 97.8) | | 92 (80.0 to 97.7) | 90 (76.9 to 97.3) |
|  |  | | |  | |  |  | |  | |  |  |
|  | |  |  | |  | |  |  | |  |  |  |

95%CI, 95% Binomial exact confidence interval

***Supplementary table iv:*** *Clinical characteristics and symptom data for participants in Round 2a and 2b.*

|  | Fortress (n=48) | Surescreen/Panbio (n=46) | AbC-19 (n=51) |
| --- | --- | --- | --- |
|  |  |  |  |
| **Comorbidities, n (%)** |  |  |  |
| Organ transplant recipient | 1 (2) | 1 (2) | 1 (2) |
| Diabetes (type I or II) | 0 (0) | 1 (2) | 0 (0) |
| Heart disease or heart problems | 2 (4) | 3 (7) | 0 (0) |
| Hypertension | 2 (4) | 2 (4) | 3 (6) |
| Overweight | 8 (17) | 8 (17) | 12 (24) |
| Anaemia | 0 (0) | 2 (4) | 0 (0) |
| Asthma | 5 (10) | 6 (13) | 7 (14) |
| Other lung condition | 0 (0) | 1 (2) | 0 (0) |
| Weakened immune | 0 (0) | 0 (0) | 1 (2) |
| Depression | 0 (0) | 2 (4) | 4 (8) |
| Anxiety | 5 (10) | 4 (9) | 2 (4) |
| Psychiatric disorder | 0 (0) | 0 (0) | 0 (0) |
| None of these | 30 (63) | 26 (57) | 29 (57) |
| **Symptoms, n (%)** |  |  |  |
| Decrease in appetite | 17 (35) | 21 (46) | 22 (43) |
| Nausea/vomiting | 6 (13) | 9 (20) | 10 (20) |
| Abdominal pain | 15 (31) | 11 (24) | 17 (33) |
| Runny nose | 13 (27) | 11 (24) | 14 (27) |
| Sneezing | 12 (25) | 10 (22) | 12 (24) |
| Blocked nose | 15 (31) | 5 (11) | 7 (14) |
| Sore eyes | 10 (21) | 7 (15) | 8 (16) |
| Loss of smell | 30 (63) | 23 (50) | 26 (51) |
| Numbness or tingling | 6 (13) | 3 (7) | 9 (18) |
| Loss of taste | 28 (58) | 20 (43) | 23 (45) |
| Sore throat | 18 (38) | 20 (43) | 17 (33) |
| Hoarse voice | 8 (17) | 9 (20) | 7 (14) |
| Headache | 29 (60) | 27 (59) | 33 (65) |
| Dizziness | 14 (29) | 8 (17) | 13 (25) |
| Shortness of breath | 18 (38) | 22 (48) | 19 (37) |
| Persistent cough | 29 (60) | 23 (50) | 19 (37) |
| Tightness in chest | 17 (35) | 15 (33) | 15 (29) |
| Chest pain | 8 (17) | 6 (13) | 9 (18) |
| Heaviness in arms/legs | 16 (33) | 20 (43) | 16 (31) |
| Fever | 30 (63) | 25 (54) | 22 (43) |
| Chills | 18 (38) | 14 (30) | 17 (33) |
| Difficulty sleeping | 12 (25) | 10 (22) | 11 (22) |
| Tired | 36 (75) | 34 (74) | 41 (80) |
| Severe fatigue | 22 (46) | 18 (39) | 20 (39) |
| Achy muscles | 25 (52) | 29 (63) | 31 (61) |
| None of these | 3 (6) | 3 (7) | 6 (12) |
|  |  |  |  |
| Results are median (IQR) unless otherwise stated. Percentages are calculated from non-missing values. | | | |
| For individuals with ongoing symptoms, symptom duration was assessed as date of symptoms start to date of visit. | | | |

| Time since symptom onset assessed for symptomatic individuals only. |  |
| --- | --- |

***Supplementary table v:*** *Sensitivity estimates of each LFIA by previous SARS-CoV-2 test result and symptom severity. FP (finger-prick, observer interpretation). Previous confirmed infection of SARS-CoV-2 refers to the occurrence of a positive PCR or formal laboratory antibody test result prior to the attendance of a research clinic appointment.*

|  | **Surescreen** | | | | **Panbio** | | | | **AbC-19** | | | |
| --- | --- | --- | --- | --- | --- | --- | --- | --- | --- | --- | --- | --- |
|  | FP sensitivity vs previous confirmed infection | | FP sensitivity vs S-ELISA/Hybrid DABA | | FP sensitivity vs previous confirmed infection | | FP sensitivity vs S-ELISA/Hybrid DABA | | FP sensitivity vs previous confirmed infection | | FP sensitivity vs S-ELISA/Hybrid DABA | |
|  | n/N | Sensitivity  (95% CI) | n/N | Sensitivity (95% CI) | n/N | Sensitivity  (95% CI) | n/N | Sensitivity  (95% CI) | n/N | Sensitivity (95% CI) | n/N | Sensitivity  (95% CI) |
| **Method of previous SARS-CoV-2 diagnosis:** |  |  |  |  |  |  |  |  |  |  |  |  |
| PCR +ve | 25/28 | 89 (71.8 to 97.7) | 25/28 | 89 (71.8 to 97.7) | 20/26 | 77 (56.4 to 91.0) | 20/26 | 77 (56.4 to 91.0) | 17/22 | 77 (54.6 to 92.2) | 17/22 | 77 (54.6 to 92.2) |
| Ab positive only | 13/16 | 81 (54.4 to 96.0) | 13/16 | 81 (54.4 to 96.0) | 13/17 | 76 (50.1 to 93.2) | 13/17 | 76 (50.1 to 93.2) | 16/28 | 57 (37.2 to 75.5) | 16/26 | 61.5 (40.6 to 79.8) |
|  |  |  |  |  |  |  |  |  |  |  |  |  |
| **Symptom Severity** |  |  |  |  |  |  |  |  |  |  |  |  |
| Asymptomatic | 4/4 | 100 (39.8 to 100.0) | 4/4 | 100 (39.8 to 100.0) | 4/4 | 100 (39.8 to 100.0) | 4/4 | 100 (39.8 to 100.0) | 3/6 | 50.0 (11.8 to 88.2) | 3/5 | 60 (14.7 to 94.7) |
| Symptomatic (any severity) | 34/40 | 85 (70.2 to 94.3) | 34/40 | 85 (70.2 to 94.3) | 29/39 | 74 (57.9 to 87.0) | 29/39 | 74 (57.9 to 87.0) | 30/44 | 68 (52.4 to 81.4) | 30/43 | 70 (53.9 to 82.8) |
| Symptomatic (mild) | 7/10 | 70 (34.8 to 93.3) | 7/10 | 70 (34.8 to 93.3) | 4/9 | 44 (13.7 to 78.8) | 4/9 | 44 (13.7 to 78.8) | 6/8 | 75 (34.9 to 96.8) | 6/8 | 75 (34.9 to 96.8) |
| Symptomatic (moderate) | 14/16 | 88 (61.7 to 98.5) | 14/16 | 88 (61.7 to 98.5) | 11/16 | 69 (41.3 to 89.0) | 11/16 | 69 (41.3 to 89.0) | 9/17 | 53 (27.8 to 77.0) | 9/16 | 56 (29.9 to 80.2) |
| Symptomatic (severe – non hospitalised) | 10/11 | 91 (58.7 to 99.8) | 10/11 | 91 (58. to 99.8) | 11/11 | 100 (71.5 to 100.00) | 11/11 | 100 (71.5 to 100.0) | 11/15 | 73 (44.9 to 92.2) | 11/15 | 73 (44.9 to 92.2) |
| Symptomatic (severe – hospitalised) | 3/3 | 100 (29.2 to 100.0) | 3/3 | 100 (29.2 to 100.0) | 3/3 | 100 (29.2 to 100.0) | 3/3 | 100.0 (29.2 to 100.0) | 4/4 | 100 (39.8 to 100.0) | 4/4 | 100 (39.8 to 100.0) |

***Supplementary table vi.*** *Sensitivity results for all antibody concentration quartiles are shown versus S-ELISA.*

| **Lateral flow immunoassay** | **Test method** | **Quartile** | **Positive vs S-ELISA, n/N** | **Sensitivity against S-ELISA, % (95%CI)** |
| --- | --- | --- | --- | --- |
| Fortress | Finger-prick, self-read | 1 (lowest) | 8/12 | 67 (34.9 to 90.1) |
|  |  | 2 | 4/6 | 67 (22.3 to 95.7) |
|  |  | 3 | 13/14 | 93 (66.1 to 99.8) |
|  |  | 4 (highest) | 12/12 | 100 (73.5 to 100.0) |
|  | Lab serum | 1 (lowest) | 11/12 | 92 (61.5 to 99.8) |
|  |  | 2 | 5/6 | 83 (35.9 to 99.6) |
|  |  | 3 | 13/14 | 93 (66.1 to 99.8) |
|  |  | 4 (highest) | 12/12 | 100 (73.5 to 100.0) |
| Surescreen | Finger-prick, self-read | 1 (lowest) | 0 |  |
|  |  | 2 | 7/10 | 70 (34.8 to 93.3) |
|  |  | 3 | 10/10 | 100 (69.2 to 100.0) |
|  |  | 4 (highest) | 16/17 | 94 (71.3 to 99.9) |
| Panbio | Finger-prick, self-read | 1 (lowest) | 0 |  |
|  |  | 2 | 8/10 | 80 (44.4 to 97.5) |
|  |  | 3 | 5/8 | 63 (24.5 to 91.5) |
|  |  | 4 (highest) | 16/17 | 94 (71.3 to 99.9) |
| AbC-19 | Finger-prick, self-read | 1 (lowest) | 8/21 | 38 (18.1 to 61.6) |
|  |  | 2 | 12/16 | 75 (47.6 to 92.7) |
|  |  | 3 | 9/9 | 100 (66.4 to 100.0) |
|  |  | 4 (highest) | 2/2 | 100 (15.8 to 100.0) |
|  | Lab serum | 1 (lowest) | 20/21 | 95 (76.2 to 99.9) |
|  |  | 2 | 15/16 | 94 (69.8 to 99.8) |
|  |  | 3 | 8/9 | 89 (51.8 to 99.7) |
|  |  | 4 (highest) | 1/2 | 50 (1.3 to 98.7) |
| Note: number of individuals tested across quartiles does not always sum to total positive on S-ELISA as a few individuals had qualitative results only. | | | | |
